# CHLA standards for library and information services in Canadian health & social services institutions 2020

**DOI:** 10.29173/jchla29526

**Published:** 2021-04-02

**Authors:** Francesca Frati, Julia Kleinberg, Lori Anne Oja, CHLA-ABSC Standards Task Force

**Affiliations:** 1 Schulich Library of Physical Sciences, Life Sciences, and Engineering, McGill University, Montreal, QC.; 2 Health Sciences Library, Jewish General Hospital, West-Central Montreal Health, Montreal, QC.; 3 Health Science Information Consortium, University of Toronto Libraries, Toronto, ON.

## Abstract

The following standards, with supporting evidence, are intended to serve as a guide to structuring minimum library services within health and social services institutions across all Canadian provinces and territories. The Standards are not intended to be aspirational. The aim of the Task Force was to ensure that the Standards update would not be so removed from the current realities and landscape that they became unattainable to many libraries. For this reason, some Standards outline requirements that are essential to the minimum function of the library, and other Standards provide recommendations only. The intended use of the Standards is to set a baseline for the provision of essential library services and resources and aid in advocating for adequate resources. It is important to note, however, that the Task Force does not intend for the Standards to prevent libraries from reaching a more advanced level of service, and we hope that in their current form they will not be a hindrance to excellence or innovation. Once published, the version of the Standards made freely available on the Canada Health Libraries Association website [1] shall henceforth and always be considered the most recent and active version of the Standards and is the version that should be used to inform practice. The Standards Standing Committee will institute a regular review and updating schedule, ensuring the currency of the Standards.

## Introduction

Informing practice with current best evidence correlates with improved patient outcomes and satisfaction, as well as quality improvement [3-10]. While it is difficult to measure direct impact on patient outcomes, and larger randomized studies are needed, a growing body of evidence shows that literature searches conducted by health information professionals can serve to improve clinical decision-making [11, 12], and clinicians report that information received by librarians or found using resources provided by the library has helped prevent adverse events, reduce unnecessary treatments or referrals [3, 12-15], inform or confirm decisions about treatment and patient management [3, 16, 17], and reduce length of stay [9, 12, 18]. According to the Medical Library Association (USA), “The health information profession provides access to and delivers important information that improves patient care and supports education, research, and publication” [19]. Information services provided by health information professionals have been shown to help mitigate barriers to use of evidence, such as lack of time and inadequate search skills, by providing mediated information retrieval, support for evidence-based practice (EBP) and information literacy (IL) [7, 8, 20-23]. This can take the form of services or Continuing Education (CE) instruction. The presence of a clinical librarian has been shown to “affect personal searching behavior as doctors were also prepared to spend longer on searches” [24]. A recent study has shown that librarian participation in clinical rounds reduces costs [21], although Madden et al [25] found that there is a “need for future research to develop standardised, validated tools that clinical libraries can use to demonstrate their financial impact.”

Health information professionals can provide evidence to support clinical governance (e.g. clinical effectiveness and research (EBP); education & training; consumer health & health literacy; staffing and staff management; using IT and information, etc.), and health governance (e.g. support for partnerships, participation and consensus; formulating policy/strategic direction; generating information/intelligence etc.) although research is needed to measure the impact of this type of support [26].

Services provided by health information professionals can also help improve research productivity and quality — in particular knowledge synthesis [27-31], and reduce waste [32-35]. This is true across healthcare disciplines, including medicine, nursing, rehabilitation etc.

Health information professionals, experts in identifying information needs and finding information to answer them, are also uniquely placed to provide continuing education and professional development instruction in evidence-based practice skills and competencies, and research shows that participants’ skills improve after receiving literature search training [3, 9, 36, 37].

There is some evidence showing that the involvement of librarians in patient- and family-centred initiatives contributes to improved patient and family experience and supports participatory care/shared decision-making and patient-centred care [38].

Contrary to popular belief, not everything is available at no cost on the Internet, and this is especially true of best evidence, which often resides behind a considerable paywall. Managing subscriptions to the necessary resources requires a healthy budget allocation and is best done by health information professionals who are able to review a variety of resources for quality, and negotiate with vendors in an informed manner using knowledge of current publishing trends in academic and healthcare along with needs assessment and benchmarking data across institutions [39]. Subscription costs increase yearly, sometimes exponentially, and the library budget allocation must adjust in order to accommodate these increases.

## Background of the Standards Update

A working group (WG) was formed that included members of the JCHLA/JABSC editorial team, and three librarians with expertise in RDM. The WG took a three-pronged approach to gathering information to develop the policy, which included reaching out to authors of previously published JCHLA/JABSC articles for feedback, reviewing existing journal data sharing policies, and holding an open stakeholder consultation webinar with the HSL community to introduce the draft policy and solicit feedback. This section will describe each of these app Hospital library standards first appeared in the 1940’s when the American College of Surgeons included minimum standards for hospital libraries in the Manual of Hospital Standardization [40]. The Canadian Health Libraries Association (CHLA) published the first Canadian Standards (last updated in 2006) in 1975 following the creation of the first Hospital Library Standards in Quebec in 1968. The Quebec standards did not get much traction at the time but did inspire a “new way to measure the information needs of a hospital [that had] taken account of the extent to which a hospital was involved in teaching various levels of personnel and had used this as the basic criterion for assigning information requirements. The new measure seemed to make it relatively easy to gauge the extent and depth of the collection needed and the type of personnel required by any given hospital library” [41].

Over the years, the Canadian, American and other health library associations in the UK and Australia have developed and periodically updated their Standards, often reviewing each other’s Standards as they evolved over time and adapting these to reflect regional practice and requirements. For the current update, the CHLA Task Force was in communication with both the MLA Standards Task Force and the Australian Library and Information Association-Health Libraries Australia group (ALIA-HLA) working on the Australian standards update, both of which were underway at the time of writing, and we have benefitted from learning about the differing approaches.

Health and social services institution library closures and consolidations occur all too frequently, along with reduced staffing, space and budgets [42-45]. Practice that is not based on evidence risks causing harm to patients [46]. A robust and evidence-based set of current Standards are an important tool for libraries to use to communicate best practices to their organization and demonstrate value. In 2016, Quebec undertook a province-wide restructuring of the healthcare system into large multi-centre healthcare networks. This restructuring had a significant impact on health institution libraries in Quebec that continues to have repercussions four years later. In the case of the Jewish General Hospital (JGH) Libraries, in Montreal, Quebec, the creation of the CIUSSS West-Central Montreal, one of the newly formed healthcare networks affiliated with McGill University, resulted in the elimination of the Chief Librarian position, ongoing changes in reporting structure and the need to expand services to the network as a whole. The responsibility to plan strategically for this expansion fell to one of the authors of the current update (Frati), and the 2006 Standards provided support for this initiative. The 2006 Standards did not include guidance for the provision of library services across library systems, so it became necessary to use the Medical Library Association (MLA) Standards for hospital libraries published in 2007, which include the relevant guidance. Another document that proved useful was the HSICT Levels of Health Library Services [47], a benchmarking tool developed by the Health Science Information Consortium of Toronto (HSICT) as part of the Library Value Toolkit [48] – see the Benchmarking tab. Used in conjunction with the two Standards, the Levels of Service document made it possible to benchmark the services offered by the JGH Health Sciences Libraries at the time. It was possible to demonstrate that services were being provided at a very high level (Gold +), and to indicate to what extent levels of service would necessarily be reduced should the library’s mandate expand beyond the hospital to the larger network without any increase to current staffing, resources and budget.

While the 2006 CHLA and 2007 MLA Standards were useful in supporting arguments for adequate staffing and resources, the need for more current evidence-based Standards was evident. To gauge pan-Canadian need for and interest in such an update, Francesca Frati and Jeanna Hough together chaired a round table discussion regarding the Standards at the CHLA conference in Edmonton in May 2017. Participants representing libraries across Canada agreed on the need for an update that could serve as a tool to help Canadian health institution libraries advocate for themselves. The association published the CHLA/ABSC Strategic Plan, 2018 – 2021 following the conference [49]. Key Strategic Direction 3: Demonstrate Value /Advocacy, includes Goal 3.1 “Review and update Standards for Library and Information Services in Canadian Healthcare Facilities.” In support of this goal, the CHLA Board approved the creation of a Standards Task Force with the mandate of updating the 2006 Standards and providing recommendations to the Board regarding long-term sustainability of the Standards.

At the same time, in late 2017, HSICT launched its three-year strategic plan. HSICT is a collective of fifty health libraries across Ontario whose purpose is to advance the role of members in health care and health education through impactful advocacy, knowledge and expertise building, and optimal resource sharing and acquisition. One strategic theme that emerged was the need to increase advocacy support to member libraries facing Ontario’s ever-challenging health care environment. A key priority in that theme was to seek collaborations with other organizations with similar goals for their members. This led HSICT to reach out to CHLA in the hopes of forming a partnership. The Standards update became the focus of this collaboration, with the levels of service document providing a benchmark for services within the Standards.

In both Canada [50] and the United States [51], recent assessment shows that hospital libraries are, for the most part, meeting but not surpassing the 2006 CHLA and 2007 MLA Standards respectively, so a complete overhaul was not deemed necessary [2, 52]. In 2019, Spencer et al. conducted a benchmarking study of hospital libraries and found that a large percentage of libraries did not have adequate staffing or budget and did not have a marketing and communications plan, or a strategic plan [44]. This confirmed the changes the Task Force proposed for the 2020 update, which emphasize the need for adequate staffing and budget, and highlighted the importance of strategic planning and assessment to show value and provide support for promotional endeavors (see Table 1).

**Table 1:** List of Standards – comparison of 2006 Standards to 2020 Standards.

2006 Standards	2020 Standards*	What’s new
**1 Administration and Organization**	**1 Administration and Organization**	No change
**2 Management**	**2 Management**	The need for either a Master’s degree or technical degree plus experience
**3 Staffing**	**5 Staffing**	Health networks New algorithm for the calculation of adequate staffing
**4 Services**	**3 Services**	This Standard outlines minimum “Bronze/Silver” level services
**5 Resources**	**4 Resources**	Consortial agreements added eResources added
**6 Promotion**	**10 Promotion and Outreach**	*Renamed* Need to show value to administration and not just promote services to users
**7 Legislation and Compliance**	**11 Legislation and Compliance**	No change
**8 Accessibility**	**12 Accessibility: Diversity, Equity and Inclusion**	*Renamed* Accessibility (web and space) for persons with disabilities Diversity and equity should be considered Access to physical space moved to Standard 9
**9 Environment**	**7 Virtual and Physical Space, and equipment**	*Renamed* Importance of virtual space Access to physical space moved here from Standard 8 Technology moved from here to Standard 12
**N/A**	**6 Professional development**	*New Standard* Recognises the need to maintain professional competencies
**N/A**	**9 Value and Advocacy**	*New Standard* Use of assessment data and evidence to show value
**See Standard 9**	**8 Technology**	*New Standard* Moved from Standard 9

* The 2020 Standards does not use the same order as the 2006, but similar Standards have been placed side by side in order to highlight what has changed and what has remained consistent.

The spirit of the Standards has historically been to provide guidance without being prescriptive and this continues to be the case. This approach allows each library to base decisions about resources and services on the current needs and strategic goals of the organization they serve, while at the same time ensuring that services are in keeping with current health library norms. The Task Force integrated recommendations coming out of the round table discussion as much as possible, including the addition of the Professional Development Standard among other changes outlined below (see Table 1). Although the format remains similar to the 2006 version, the update and development of benchmarking tools and other useful resources for putting the Standards into practice and the development of a certification process for use during Accreditation are within the mandate of a Standards Standing Committee (see methods).

The biggest changes to the Standards have been to make provisions for libraries functioning within larger networks (which at the time of the 2006 Standards were not common in Canada), to acknowledge that not all libraries may require physical space, but that all library services are in need of a strong and visible virtual space and adequate technology. Another important change is a move away from marketing and promotion alone, to a focus on the use of assessment data and evidence to show value and to advocate for library services. As well, the 2020 Standards use a new and improved staffing algorithm, and recognise the importance of professional development, as well as diversity, equity and inclusion as important considerations. The 2020 Standards refer to the HSICT Levels of Library Services tool “Bronze/Silver” [47] representing an example of minimum library services, which are both advisable and attainable, in libraries across Canada. The name of the Standards was revised to include social services as in some Canadian provinces and territories, health and social services are provided as part of integrated systems of care.

To this end, the Task Force added several new Standards, and renamed several others.

## Methods

The JCHLA/JABSC Data Sharing Policy asks authors of research articles and program descriptions to make the data associated with their submitted manuscript available in a public repository or as part of the manuscript (e.g., as a supplementary file). Manuscripts are to include a Data Availability Statement (DAS) describing where the supporting data for the article can be found, including hyperlinks to publicly archived datasets that were analyzed or generated during the study. Manuscripts will be required to have a DAS, regardless of whether the data can be made publicly available, whether access to the data are restricted, or whether, in the case of a Program Description, there are no additional data beyond those reported with the manuscript. Full details of the criteria necessary to write a DAS are included in the Data Sharing Policy that is available on the JCHLA/JABSC Editorial Policies webpage.xxx Rather than convene a large task force to review and update the Standards, as had previously been the case, a relatively small task force comprised of three health information professionals, with experience in hospital libraries and consortial management, co-authored a new draft of the Standards, undertaking literature searches to identify evidence in support of the revised Standards. After copyediting, the initial draft of the update (including new and revised Standards) was subsequently put through a series of expert peer reviews, followed by a member consultation. The Task Force recommended that a CHLA Standards Standing Committee (SSC) be formed so that upon completion of the current update the SSC would have as its mandate to update the Standards on a regular basis. In order to prevent the Standards from becoming quickly outdated, the Task Force recommended that the document henceforth exist in the form of a living document that remains consistently relevant to current best practice and takes into account evolving standards and practices in health care and libraries. The Board approved a motion to create the SSC in early 2020, and the committee had their first meeting on February 24, 2020.

The Task Force submitted the final manuscript for publication in the April 2020 issue of the Journal of the Canadian Health Libraries Association. The accepted manuscript was made available to members via the CHLA website in December 2020, and the newly formed SSC will review the document on a regular basis, and update it as necessary based on any newly identified evidence.

The following stakeholders participated in the aforementioned series of expert peer reviews:

1. CHLA Board
2. Two information professionals with experience in managing services in a library system within a health care network or provincial library system: Tim Tripp, Director of Library and Information Services at the University Health Network in Toronto, and Susan Baer, Transition Lead – Health Sciences Library Regina General Hospital. Susan Baer subsequently wrote Appendix 3 Considerations for Library Services within Provincial Library Systems.
3. HSICT Management Committee
4. Standards Standing Committee
5. Fédération des milieux documentaires: section santé et services sociaux (FMD3s - Quebec Chapter of CHLA)
6. Member consultation

After each stage of expert peer review, the Task Force reviewed feedback and made revisions. This approach to arriving at consensus allowed the initial draft to be written over a relatively short period, while at the same time ensuring that a large number of subject experts contributed to the content. The Task Force held a members’ consultation to secure buy-in from members of the association and expand the pool of expertise contributing to the Standards. In response to the feedback received, the Task Force added several appendices to the Standards:

- Appendix 2 Considerations for Library Services within Health Care Systems or Networks
- Appendix 3 Considerations for Library Services within Provincial Library Systems
- Appendix 4 Staffing within Library Systems/Networks or in Libraries Providing an Advanced Level of Service
- Appendix 5 Additional Considerations for Libraries Providing an Advanced Level of Service

To ensure the rigour of the current Standards and identify best evidence, the Task Force planned and oversaw sixteen rapid scoping reviews in collaboration with the CHLA Research Committee and the CHLA Knowledge Synthesis Interest Group. The Task Force identified between one and two relevant search questions for each Standard and comprehensive rapid scoping searches were performed in one or two appropriate bibliographic databases by volunteer expert searchers from across Canada (the search strategies and full results will be made available on the CHLA website). Due to the rapid nature of the searches, we allowed searchers to request peer review of their search(es) at their discretion. Because the expert searchers possessed subject expertise, they were also tasked with screening for relevant articles with the goal of identifying either: 1) evidence that supported the proposed Standard, or 2) evidence that refuted or suggested changes to the proposed Standard.

Screening was done iteratively in two stages: at stage one, each searcher did an initial screening, identifying relevant articles based on relatively broad inclusion/exclusion criteria and using the text of the Standard itself as additional criteria for determining relevance; at stage two, the Task Force member responsible for each Standard reviewed the results of the initial screening and provided feedback to the searcher, providing the searcher with a sample of relevant articles the searcher then used to conduct a second, more targeted screening. The searchers then provided the Task Force with a small set of highly relevant articles for the Task Force to read and integrate into the Standards as appropriate. Each search was set up as an alert and searchers will submit any new evidence retrieved to the SSC for a period of one year. At the end of this first year, the SSC will take over management of the search alerts and schedule updates to the Standards according to a predetermined schedule.

### Limitations

It is important to acknowledge certain limitations. Due to constraints (time, human resources) the Task Force considered that conducting a series of full scoping reviews to support the Standards was not feasible and therefore, used a rapid review approach. The rapid nature of the searches, screening and integration of evidence has introduced the possibility of bias into the Standards. Bias is also possible due to the fact that the Task Force identified search questions based on the proposed new Standards, rather than using the results of a knowledge synthesis to inform the proposed changes. The Task Force felt, and the CHLA Board agreed, that Standards based on practitioner expertise, supported by a series of expert peer reviews, followed by a series of rapid comprehensive searches, a members consultation, and subsequently, a continual update of the Standards according to a regular schedule, would be sufficient to develop the Standards and identify best evidence, and would serve to mitigate potential bias.

As well, it is important to note that for some aspects of the Standards, the Task Force did not identify any existing evidence, in which case we deemed expert opinion the highest level of evidence available. We consider that basing these aspects of the Standards on a consensus of experts was preferable to excluding mention of these aspects due to lack of evidence. For example, the HSICT Levels of Library Service guide was developed as a benchmarking baseline which has proved useful to position the current Standards. The Standards Standing Committee has been given the mandate to work with the HSICT to review and update the Levels of Library Service, originally published in 2016; to produce an expanded, versatile tool to support library benchmarking activities; and to update the Standards accordingly.

Exemptions for sharing data will be made in rare cases where de-identified data cannot be shared due to their proprietary or sensitive nature (e.g., Indigenous data subject to the OCAP principles [24,25], confidential financial information from vendors) or when research projects were initiated before 2021 and did not receive consent from participants to share data. Authors are still required to provide a DAS in such cases, explaining why the data cannot be shared.

The JCHLA/JABSC Data Sharing Policy defines data as the materials collected and reported as evidence for the results or outcomes in either a research article or program description. Data formats may include (but are not limited to) spreadsheets, text files, interview recordings or transcripts, images, videos, outputs from statistical software, or computer code or scripts. Authors are encouraged to save their data in open data formats.

Authors are also encouraged to share accompanying documentation of the data (e.g., data dictionaries, codebooks, readme files) to facilitate the understandability and reusability of the data. Measures should be taken to de-identify data to protect the identity of research participants (see the Data Sharing FAQ page on the JCHLA/JABSC website for guidance).

The JCHLA/JABSC Data Sharing Policy provides a list of recommended repositories where authors can share their data and provides guidance to help authors decide about where best to share. Additionally, guidance on how to choose a license to apply to research data has also been included.

## Standard One: Administration and Organization

### Background

The Accreditation Canada [53] Leadership Standard 5.3 requires that the organization make education and reference materials and research information available to staff, users and families, while Standard 5.4 requires that:

- Systems must be in place to provide clear direction and timely access to education, reference and research materials that have been evaluated against current and future needs.
- A method for linking to relevant external databases, information networks and bodies of research knowledge must be provided.

### Expectations

The Library and Information Service must be positioned to communicate and collaborate with decision makers throughout the organization [54], including, but not limited to, human resources, quality improvement and accreditation, continuing education and high-level committees. The library manager should have control over a dedicated budget allocation managed by a qualified Health Information Professional who reports to senior management. The library manager should have overall responsibility for library management activities and decision-making related to the strategic planning, facilities, human resources, and service provision [2].

This allows for ongoing assessment of the priorities of the organization, which informs the development and implementation of appropriate services to meet these needs and to maintain alignment with the organization’s mission, vision, goals and strategic plan [55].

## Standard Two: Management Administration and Organization

### Background

Managing a health library requires specific knowledge and skills related to library and information management, and additional knowledge, skills, and flexibility related to understanding and meeting the rapidly evolving needs of users in a healthcare setting [56]. A demonstrated ability to lead “others to define and meet institutional goals” [57] is also of vital importance in libraries with staff. Critical areas of expertise include “planning, organizing, controlling, staffing, budgeting, facilities management, automation, and coordination/integration” [58].

### Expectations

A health information professional has earned a master’s degree from a program that is accredited by the American Library Association (ALA) or is recognized by either the ALA or an appropriate national body. Health Information professionals without master’s level education (e.g. library technicians) may serve in a managerial capacity for libraries offering bronze level services (see Bronze/Silver *HSICT Levels of Library Services)* [47], and must have a Library & Information Technology Diploma from a recognized college, a minimum of five years of progressive experience in a health library and a minimum of two years proven leadership experience.^1^ Library manager duties and responsibilities vary across institutions. The library manager should be involved in the development of the job description and participate in the hiring process potential successors and should work with human resources to ensure the library manager job description evolves over time and continues to reflect the necessary competencies.

Competencies for health information professionals at the bronze service level include an in-depth knowledge of print and electronic information resources, as well as the design and management of effective and efficient information services that reflect the strategic goals of the organization and its users [4]. Health information professionals should have advanced or expert level knowledge in most of the competencies highlighted in the MLA Competencies for Lifelong Learning and Professional Success 2017 [57]. Where library and information centres have resources to support silver and gold level services, health information professionals should also be further developing their expertise in knowledge and intellectual capital management for their institutions [59].

See also Standard Six.

## Standard Three: Services

### Background

Methods of delivering evidence-based practice are constantly changing. The health information professional must continually evaluate these new methods to ensure that the services offered by the library reflect the needs of its user groups [60].

### Expectations

The health information professional conducts an ongoing assessment of the information service needs of the organization, and uses this assessment to develop and implement appropriate services to meet these needs and to maintain alignment with the organization’s mission, vision, goals and strategic plan.

The minimum level of library services must include but need not be limited to (see Bronze/Silver *HSICT Levels of Library Services* [47]:

- Reference services, i.e. personalized assistance provided to library users either in-person or virtually, including library orientation.
- Literature searching and search alerts.
- Scholarly communications support e.g. citation support, impact factors.
- Interlibrary loans (ILL).
- Evidence-based practice/Information literacy training (e.g.: question formulation, literature searching, levels of evidence etc.).
- Provision of access to and/or maintenance of searchable catalog/index of library resources.
- Development and maintenance of a library online presence (whether inter- or intra-net).
- Identification of copyright best practices.

## Standard Four: Resources

### Background

Resources include current authoritative collections of print, electronic resources (eResources) and multimedia resources that support the timely provision of evidence-based practice. As technology advances, subscription costs continue to increase [61, 62], and budgets are not always commensurate with the size of the organization [44], so health libraries must move from *Holdings* (“Just in case”) to *Access* (“Just in time”) strategies.

The Library and Information Service’s primary task is to evaluate, select, maintain and provide access to relevant information resources that support all user information needs, which could include patient care, education, administration, research, legal, consumer health, and outreach programs [63]. The health information professional will optimize the value of these resources to the organization by:

- Improving user access.
- Sharing resources.
- Creating effective partnerships.
- Addressing economic issues.
- Negotiating database license agreements.
- Leading or influencing Request for Proposal (RFP).

### Expectations

The health information professional uses a variety of tools and expertise, both formal and informal, to assess the evidence-based resource needs of medical staff and healthcare personnel [42]. These could include:

- A collection development policy that serves as a framework to support selection of materials by considering the goals and objectives of the organization, the priorities of different activities, the needs of clinicians, researchers and administrative staff, and budget allocation [63]. This policy also helps communicate how the library makes resource decisions to library users and further promotes the value of the library [64].
- Resource analysis to ensure best possible access to evidence via either individual subscription or collaboration with consortia; library resource sharing (i.e. ILL, document delivery), access, and agreements that enable the efficient provision of materials not available onsite.
- Membership in library and information consortia that increase access to quality evidence-based information cost-effectively by utilizing consortial discounts and licensing services.
- Effective access to the resources onsite and remotely (See *Standard 7*).

## Standard Five: Staffing

### Background

An appropriately staffed, and highly skilled library staff are required to meet the information needs of an evidence-based environment. Demand for information and evidence-based competency instruction, as well as the ratio of Librarians to Library Technicians is driven by the size and complexity of the institution, as well as factors such as:

- The level of service provided.
- The number of medical residents, undergraduate medical students and other program interns at the facility.
- The geographic size of the region. Fully integrated health region libraries provide services not only to acute care facilities, but also to long term, continuing care and home care, primary healthcare including mental health and addictions, community health and emergency medical services, often across broad geographic areas.
- Partnerships both within and outside the organization/institution (for example, consortial relationships).

### Expectations

No staffing formula can account for the variations in institutional size, vision, mission, and user needs. In order to offer minimum service levels, the Library and Information Service uses the following formula as a guide (see Appendix 1 for full formula):


$$\sqrt{\text{total FTE institution}}/\text{ 16}.\text{18}0\text{3399}$$

The Van Moorsel formula is the only validated formula available at the time of writing, and provides an easy method to calculate staffing ratios. This formula is based on benchmarks across American institutions, but can equally be applied to Canadian Institutions. The formula uses a sliding scale which “allows the library staffing standard to be driven in dynamic relation to organizational size, rather than by a fixed denominator” [51].

The staffing grid in Table 2 provides an overview of minimum staffing for basic services as calculated using the formula across a sample of institutions by size, and can be used as a general guide to staffing for libraries of various sizes.

**Table 2:** Staffing grid for libraries providing minimum services.

Number of institution FTE*, ^†^	Number of FTE health information professionals
400	1.24
625	1.55
900	1.85
1225	2.16
1600	2.47
2025	2.78
2500	3.09
3025	3.40
3600	3.71
4225	4.08
4900	4.33
5625	4.63
6400	4.94

* calculated using square roots in increments of 5 from 20 to 80 (20 x 20= 400, 25 x 25= 625 etc.)

† In addition to employees in all entities which the library serves, Total Institutional FTE includes all active medical staff, as well as healthcare personnel under service contracts, regardless of whether these individuals are technically considered institutional employees.

Having completed the calculation,

- If the result is less than or equal to 1 FTE, some percentage of the 1 FTE must be a health information professional with managerial experience. The health information professional may be through contract, outreach or a joint venture. Contingency staffing may be used to address fluctuations in the need for specialized services [65].
- If the result is 1-3 FTE, then 1 FTE must be a librarian or paraprofessional with managerial experience.
- If the result is greater than 3 FTE, 33% must be librarians and the service/department manager must be a librarian or library technician with managerial experience [66].

The remaining percentages should be a mix of qualified library technicians, assistants, clerks, and skilled volunteers. A limitation of the Van Moorsel formula is that it does not account for patients and families as a user demographic: there may be different staffing considerations when providing services to patients and families. Volunteers should not be used in place of professional staff, or to justify staffing levels that do not comply with the above staffing formula [67].

Regardless of the service model (a single library serving the entire network, individual institutions served by multiple networked libraries), the number of FTE library staff should be calculated using the algorithm as a guide but should additionally take into consideration the number of locations where services are delivered and if any advanced services are being provided that require additional capacity.

When a large system is served by multiple networked libraries, it is advisable to have one FTE dedicated systems health information professional to manage a team that supports the technical services functions such as the catalogue and circulation system, the website and proxy resolver etc., thus ensuring consistent service and reducing duplication of effort across the system. If a decision is made to maintain and staff libraries at multiple locations, a level of staffing above that specified in the library staffing formula will be necessary to the extent that:

- tasks will be duplicated and
- the time of the staff will be used to travel among institutions.

## Standard Six: Professional Development

### Background

The quality and effectiveness of library and information services depend on the expertise of staff. Rapidly evolving needs of healthcare communities, changing technologies, and growth in professional knowledge require health information professionals to expand their knowledge and update their skills on an ongoing basis [68]. As a result, the organization responsible for providing information services to its health professionals is also responsible for enabling library staff to maintain competencies outlined by the relevant library associations and continue to learn [57, 69].

### Expectations

Health information professionals are responsible for identifying and pursuing continuous learning that improves knowledge and skills. Employers are responsible for providing adequate support for continuing education of their health information professionals.

The health information professional and the organization will partner to show commitment to learning as guided by International Federation of Library Associations and Institutions (IFLA) Guidelines for Continuing Professional Development: Principles and Best Practices [69], summarized below:

- Conduct a regular needs assessment based on reviews of employees’ performance in relation to the institution’s mission and goals, resulting in learning plans for both individuals and staff as a whole.
- Identify a broad range of learning opportunities, both formal and informal, and in a choice of formats that maximize investment and access to quality continuing education.
- Maintain consistent documentation of an individuals’ participation in learning.
- Endeavor to invest a minimum of $886/individual annually staff development which is consistent with industry and non-profit investment averages [70].
- IFLA guidelines suggest approximately 10% of work hours provided to professionals for attendance at workshops, conferences, in-service training, and other educational activities, as well as for informal learning projects, including professional association and research work, taking into consideration relevant collective agreements and terms of employment. However, industry standards generally show that time allowed for Professional Development is roughly 45 hours annually [71].
- Provide periodic evaluation of the results of staff development and its impact on its users.

See also Standard Two.

## Standard Seven: Virtual and Physical Space

### Background

As clinical decisions are often made outside of normal working hours, evidence-based practice resources must be accessible at all times. As evidence-based resources are increasingly available exclusively online, and libraries and library networks must serve institutions across large geographic areas, the virtual library space has become a necessity for health and social service institution libraries and must be robust enough to serve as the access point for information services and resources [72]. At the same time, health information professionals are increasingly embedded within healthcare services and departments and research teams. For this reason, user-centred services do not necessarily depend on the traditional library space [73]. However, the need for physical space may remain, and a decision about whether to have one must depend on close consideration of the needs of the institution and its users, as well as meeting the requirements of affiliation agreements in the case of teaching hospitals and health and social services institutions. The use of physical space should be assessed on an ongoing basis in collaboration with key stakeholders and may evolve to include functions other than traditional library functions.

### Expectations

#### Virtual Space

The Library and Information Service has an appropriate environment for delivering access to evidence-based practice. The library’s digital systems and online presence should be maintained and managed primarily by library staff in coordination with organizational IT departments in order to ensure optimal decision-making and timely maintenance. Design of virtual spaces should consider the local needs of the institution and ensure long term sustainability [74]. Virtual spaces should be given prominent placement, such as a position on the main navigation within the institution’s intranet and/or internet [72, 75]. A list of hospital library staff and their roles should be easily discoverable on the institution’s website. The Virtual library space is managed and populated by health information professional(s) and is capable of hosting content related to information retrieval, use and management, and access to online evidence-based resources such as biomedical databases (e.g. Medline, CINAHL, etc.), point of care tools (e.g. UpToDate, DynaMed etc.), consumer health and patient education resources (e.g. MedlinePlus, in-house patient education materials). Additional subscriptions to products or tools may be required if proxy server or Virtual Private Network (VPN) capability is necessary to provide off-site access (see Standard Four and Standard Eight).

#### Physical Space

The need for physical space may be a requirement according to the needs of the institution, and the services and resources the library is expected to deliver and/or house, for example services to patients and families, institutional archives, etc. University affiliation agreements should be consulted as physical space with 24/hour access may be a requirement [76].

Physical space accommodates current and future (three to five years) requirements, connectivity for computers and/or laptops, print collections, staff workspaces and meeting rooms, as well as areas for quiet study and group meetings [6, 77]. Library staff should work with the institution’s security department to ensure the safety of staff, users and equipment.

Whether or not the library exists as a physical space, health information professionals should have an appropriate working environment that includes private space for meetings and phone calls, and access to appropriate equipment and technology (see Standard Twelve). Facilities and equipment for instruction and workshops are available for use by library services (see Standard Eight).

## Standard Eight: Technology

### Background

Whether the library space be physical, virtual, or both, technology is essential to the functioning of libraries and serves as the foundation for the provision of services (including literature searches and EBP/IL instruction) and access to information [78]. As technology increases in sophistication and is updated more frequently, libraries must have budget allocation and infrastructure to be able to keep pace with evolving needs and technological advances [76, 77]

### Expectations

The library maintains and regularly updates software, systems and technology to meet the needs of its users and to enable the effective provision of services and access to resources. The library controls unique technology related to library services (e.g. Integrated Library System (ILS)) and is consulted on institutional IT decisions that impact those library systems (e.g. authentication and security). Specialized software and tools are needed by the library to maximize ease of access for the library’s clientele (link resolvers, database management systems, as examples). The library/information service works in partnership with the institutional IT department in order to purchase, install and maintain library software and technology [79].

## Standard Nine: Value and Advocacy

### Background

Studies have established that library services add value in a healthcare setting by positively impacting patient care [7], and improve clinician decision-making [8, 9, 11].

### Expectations

The library/information service uses evidence to demonstrate the link between services and resources and patient care and safety, patient education/consumer health & health information literacy, quality improvement indicators, health professional education, and other important institutional functions [80]. This relationship is communicated effectively to upper management and stakeholders. Libraries can communicate this value through various means, for example:

- Demonstrate value in terms of outcomes rather than output; for example, annual reports should highlight projects that support institutional objectives [4].
- Collection and dissemination of statistics is done with illustration of value in mind; for instance, validated impact measurement surveys (Recommended: Quick assessment tool validated by Farrell and Mason [13], whose results can be compiled by one or more institution to provide objective evidence of the benefits of library services, are used. Documentation of libraries’ contribution of evidence relating to patient care decisions (e.g. “clinical health information professional” services, provision of literature related to specific cases) is provided.
- Library staff represent the library by participating in committees related to accreditation, quality improvement, patient safety, patient education, and/or professional development in order to raise visibility and awareness of library issues and services. Library provision of information related to these activities should be documented and disseminated (see also Standard Six).
- Qualitative and quantitative evidence should be carefully integrated to tell the impact story [24, 81, 82].

## Standard Ten: Promotion and Outreach

### Background

Health Libraries have a history of involvement in patient education, general literacy and information literacy efforts and are an excellent resource in advancing health literacy practice and research [83]. The library publicizes services and resources to increase user awareness and encourage efficient use of the services and resources that are available. Effective library promotion educates users about databases and available resources, and eliminates barriers, encouraging the increased use of evidence-based information in treatment decisions. The library proactively identifies and leverages user needs, and proactively reaches out to users to determine how these needs can best be met [42]. Prominence on the institutional website helps promote library services and resources and demonstrate value [75].

### Expectation

The library and information service actively promotes evidence-based practice services to user groups, whether they are within or outside the institution. Promotion activities may utilize both traditional and non-traditional means such as the classic elevator speech or social media to reach users [84].

The library and information service bases its promotion strategy on data collected through needs-assessment surveys and on institutional objectives and strategy. Planning service assessment and careful outcome evaluation will strengthen the ability to identify best practices and increase effectiveness of health information outreach [85]. Outreach activities should be directly linked to specific goals and needs. Ideally, promotion objectively demonstrates the evidence linking use of library resources and services with desired outcomes for the institution (see Standard Nine) [86].

## Standard Eleven: Legislation and Compliance

The library and information service complies with relevant legislation and provincial health information protection acts (e.g. copyright), accreditation, affiliation agreements, and organizational policies, procedures, standards and relevant collective bargaining agreements and terms of employment.

## Standard Twelve: Accessibility: Inclusion, Diversity and Equity

Health information professionals shall respect the history, culture and values of their colleagues, coworkers and major user groups and endeavour to provide access to information resources and deliver services that meet the inclusive needs of their community. This includes adequate provisions to ensure that information resources are physically and virtually accessible to users with disabilities. These principles should also be factored into hiring decisions.

Libraries should refer to any provincial or regional legislation with regard to accessibility as well as any national calls to action to address cultural and racial inequalities (e.g. Truth and Reconciliation Commission). Helpful examples of standards and guidelines in research libraries can be found at:

- Association of College and Research Libraries (ACRL). *Diversity Standards: Cultural Competency for Academic Libraries* (2012) http://www.ala.org/acrl/standards/diversity
- Association of Research Libraries. *Diversity Equity and Inclusion* (2018) https://www.arl.org/focus-areas/diversity-equity-and-inclusion#.XNBxcK-0URZ

CFLA/FCAB *Truth and Reconciliation Report and Recommendations* (2017) http://cfla-fcab.ca/wp-content/uploads/2017/04/Truth-and-Reconciliation-Committee-Report-and-Recommendations.pdf Methods of delivering evidence-based practice are constantly changing. The health information professional must continually evaluate these new methods to ensure that the services offered by the library reflect the needs of its user groups [60].

## Footnotes

1 We provide these guidelines based on the common requirements found in library manager job descriptions.

## Appendix 1: Explanation of Golden Ratio Used in Staffing Formula

Use of the golden ratio, rather than an arbitrary FTE, allows “the library staffing standard to be driven in dynamic relation to organizational size, rather than by a fixed denominator. Further, the incorporation of … ‘the golden ratio’ harmonizes the library staffing standard in geometric symmetry with organizational size of the parent institution” [51] providing a more realistic calculation, as compared to the previous standard which was calculated as follows: total institution FTE/700 = minimum library FTE. In contrast to the Van Moorsel formula, there is no information regarding whether and how the previous staffing formula was developed and validated. At the time of writing no other evidence-based validated staffing formulas have been identified.

$$\begin{array}{l} \sqrt{\text{total FTE institution}}/10\left(1.60183399\right)=\text{FTE health information professionals}\\ \text{FTE=full-time equivalent}\\ 1.60183399=“\text{the golden ratio}” \end{array}$$

## Appendix 2: Considerations for Library Services Within Healthcare Systems or Networks

An increasingly large number of Canadian health and social service institution libraries must provide services to a system or network of health and social services institutions. These standards do not attempt to dictate a single manner in which services must be provided throughout a system. Rather, they provide guidelines based on the amount and the nature of services and staffing that must be available for the system as a whole.

“A hospital system can provide [Evidence-based practice (EBP)] services and resources for its affiliates in several ways:

- Each affiliate hospital may maintain a separate library.
- Services and resources may be provided from a central location.
- Support staff may be present at each location, with professional services provided centrally.
- Support staff may be present at each location, with a circuit health information professional arrangement.
- There may be a hybrid system, in which arrangements differ among affiliates.
- There may be coordinated resource sharing among the libraries.
- There may be a substantial network of electronic resources available to all affiliates" [2].

The structure of the library’s system and the level of service provided will depend on the location of existing physical collections and staffing, emerging needs based on the institution’s development and growth, the number and complement of library staff, what new initiatives the institution requires, and what mid-level management can be resourced within the library system.

Depending on a host of factors, including physical proximity of the affiliates and the extent of electronic access to resources, the health information professional and health system administration will collaborate in making decisions about centralization or decentralization of library resources and services and extent of staffing in the libraries. If the library has a physical space and no library manager is present, there must be a librarian or library technician on-site at each location to manage the daily operations and provide minimum services. This ensures smooth flow of operations and enables users of each location to obtain assistance in finding needed information.

Each separate library location should have convenient, reliable access to a quality core collection of EBP resources, tailored to the needs of the institution. Evidence should primarily be available virtually, in the form of an electronic collection. Ideally the electronic collection is accessible both on-site and remotely. The electronic collection may be supplemented by materials (books and articles) borrowed by the library via interlibrary loan, as well as a curated in-house print collection. An arrangement whereby print materials are physically housed in a central location and transmitted to other locations on demand (by email or other means) is an option.

## Appendix 3: Considerations for Library Services Within Provincial Library Systems

### Background

In the past decade, several Canadian provinces have transitioned to provincial health systems, removing regional health boundaries. Their goal is to provide more streamlined patient care, better coordinate healthcare services across the province, and to improve access to care regardless of location. Provinces that have made the administration and structural changes to their health systems aim to standardize services.

### Expectations

For provinces that have adopted provincial health authorities, and are considered a single system, it follows that the library and information services delivered within that health system will also be merged, with the goal of providing a minimum standard level of service to all users. The Standards outlined in the previous pages certainly apply to a provincial library service. There may be specific details added to qualify the standardization.

Standards one and two are relevant in a provincial library system. It is important that the head of the library system be qualified information professional who understands all aspects of the library and its services. Provincial health authorities should avoid hiring clinical or non-information professional administrative staff to lead their library systems.

### Standard Three: Services

Common procedures and policies across libraries in the provincial system should be put in place in order to provide a similar experience for all library users, and to ensure minimum services across the system. Services and delivery should be based on evaluation of client needs. This will also help define what role the library plays in supporting information to patients and their families. Should higher level services be needed or desired, either across the network or in individual institutions within the network, additional staffing, resources and attention to core competencies is necessary, as it is for individual libraries or libraries within non-provincial systems or networks (see appendices 4 and 5).

Ensuring that consistent, high quality minimum services are delivered regardless of location or clinical status involves analysis of existing systems and on defining the types of user served, and standardizing teaching materials, collection resources, and websites, in order to ensure easy access to the library’s resources and services.

The provincial library serves as the primary department within provincial health authority for the organization, accessibility, and discoverability of knowledge-based information, whether internal or external information sources.

The provincial library may be involved in the organization and development of patient and public content for the provincial institution, as specialists in information literacy and health information literacy.

The provincial library will need to achieve internal depository status for its institution and define the types of publications that should be collected and retained, regardless of format, and collaborate with the records management department to coordinate efforts and repositories. The provincial library must use its expertise to improve the accessibility of provincial governance documents related to the provincial healthcare system, including policies, reports, and commissioned reports, by collaborating with the departments responsible for creating the documents.

### Standard Four: Resources

The library must have the authority to select, evaluate, acquire, and establish access to online information resources and healthcare evidence for the provincial health authority. The library must lead the development of and own the provincial collection development policy, consulting the appropriate clinical disciplines to balance content, and establishing a robust evaluation process, as in Standard four. Most provincial healthcare systems have a centralized contracting and procurement department which would take primary responsibility for this, however this must be informed by the expertise of the library.

## Appendix 4: Staffing Within Libraries Providing an Advanced Level of Service

The amount of staffing throughout the system should be at least at the level specified in the library staffing formula for minimum i.e. Bronze/Silver level of service, taking all components and needs of the healthcare system into account. Whether each hospital or institution is treated separately in determining staffing levels, or the system is taken as a whole, is left to the judgment of the health information professional and administrators. The important point is that staffing is sufficient to serve the number of users, and is appropriate for the level of service required to meet the needs of the organization. It stands to reason that libraries providing services that are at Gold level, such as support for systematic reviews and other knowledge synthesis, or research data management etc. (or higher in the case of libraries providing services not listed in the HSICT document), will require additional staff.

The grid in Table A1 may be used to determine appropriate staffing needed by level of service [47].

**Table A1:** Staffing grid by HSICT level of service.

Number of institution staff*		Number of FTE health information professionals		
		Bronze	Silver	Gold
20^†^	400	1.24	1.55	1.85
25	625	1.55	1.93	2.32
30	900	1.85	2.32	2.78
35	1225	2.16	2.70	3.24
40	1600	2.47	3.09	3.71
45	2025	2.78	3.48	4.17
50	2500	3.09	3.86	4.63
55	3025	3.40	4.25	5.10
60	3600	3.71	4.63	5.56
65	4225	4.08	5.02	6.03
70	4900	4.33	5.41	6.49
75	5625	4.63	5.79	6.95
80	6400	4.94	6.18	7.42

* Includes all active medical staff, as well as healthcare personnel on service contract

† Calculated using square roots in increments of 5 from 20 to 80 (20 x 20 = 40, 25 x 25 = 625 etc.)

The following formulae are used to calculate number of library staff needed per level of service:

$$\begin{array}{l} \text{Bronze }\sqrt{\text{total FTE institution}}/10\left(1.61803399\right)\\ \text{Silver }\sqrt{\text{total FTE institution}}\times 1.25/16.1803399\\ \text{Gold }\sqrt{\text{total FTE institution}}\times 1.5/16.1803399 \end{array}$$

## Appendix 5: Additional Considerations for Libraries Providing an Advanced Level of Service

Advanced levels of service may include the following:

- Advanced research support such as systematic and scoping reviews
- Consults on research strategies and grant applications
- In depth information literacy training including customized training modules
- Customized content delivery including app development
- Research Data Management
- Organizational record management or Knowledge Management

Regardless of the service model (a single library serving the entire network, individual institutions served by multiple networked libraries), when an advanced level of service is requested to meet the needs of the organization being served, i.e. Gold level of service or higher, the following should be considered in addition to level of staffing (number of FTE library staff):

### Competencies (also see Standard Four)

It is important to ensure that library staff have the required skills and competencies that will enable them to provide services that are above the minimum. Indeed, advanced levels of service cannot be provided in the absence of the required skills and competencies. When hiring decisions are made, core competencies appropriate to the highest degree attained (whether master’s or technical) must be taken into account along with years of experience and any additional certificates received, and must be reflected in the compensation.

Additionally, many higher-level skills and competencies (for example expert searching in support of systematic reviews and other knowledge syntheses) are not typically obtained during the course of study, as most information management programmes are generalist in nature. Lifelong learning is essential not only to obtaining specialized skills, but also to keeping up with advances in healthcare and health information management. Ensuring that staff have professional development and continuing education opportunities is therefore particularly important if a higher level of service is required.

### Resources (also see Standard Six)

Access to appropriate evidence-based resources (bibliographic databases, point of care tools, full text of journal articles etc.) is essential to evidence-based practice and to the provision of higher levels of service. Institutions requiring higher than minimum service must ensure that adequate funding is available to the library for subscriptions to the necessary resources and collections.

While membership in the Canadian Medical Association provides access to selection of resources to physicians, access to such resources through association membership is not consistent for nursing and multidisciplinary staff. Researchers working in health and social services institutions and who are not faculty at a university do not typically obtain access to resources through such memberships. University library licensing agreements do not allow for the provision of access to healthcare staff at affiliated institutions, unless staff have a cross-appointment, therefore university resources should not be considered a solution to the problem of access to best evidence.

## Appendix 6: Glossary

**Advanced library services:** Services that require additional staff capacity and master’s level skills sets including systematic and scoping reviews, Research Data Management, information literacy training and learning tool development, and customized content delivery including app development.

**Budget:** The amount of money that a department or institution actually uses, which may be higher or lower than its budget allocation.

**Budget allocation:** The amount of money earmarked for a department or institution to spend for a specified time period or purpose.

**Consortia:** A group of libraries that have formally agreed to the cooperative sharing of resources and/or combined purchasing power. Plural of consortium.

**Consumer health information:** “Information intended for potential users of medical and healthcare services. There is an emphasis on self-care and preventive approaches as well as information for community-wide dissemination and use [87].

**Core collection:** A minimal collection of current and authoritative information resources in any given field or for a particular type of library.

**Document delivery:** Provision of library and information resources requested by users. Document delivery may include circulation, photocopy services and interlibrary loans

**eResources:** A range of information found online and can include journal articles, newspapers, books and data.

**Evidence-based practice (EBP):** Evidence Based Practice (EBP) relies on scientific evidence for guidance and decision-making. It includes systems, resources and services to help health professionals acquire the knowledge and skills needed to maintain and improve competence, to support clinical, managerial and business decision making, to support performance improvement and activities to reduce risk to patients, and to satisfy research needs.

**Health and social services:** In some Canadian provinces and territories, health and social services are provided as part of integrated systems of care [88].

**Health information literacy:** “the set of abilities needed to recognize a health information need, identify likely information sources and use them to retrieve relevant information, assess the quality of the information and its applicability to a specific situation, and analyze, understand, and use the information to make good health decisions” [89].

**Health information professional:** “The health information profession provides access to and delivers important information that improves patient care and supports education, research, and publication” [19]. A health information professional has earned a master’s degree from a program that is accredited by the ALA or is recognized by either the ALA or an appropriate national body, or holds a Library & Information Technology Diploma from a recognized college. A health Information professional should also possess knowledge of health information resources and have experience within a health library environment.

**Information literacy (IL):** From the ALA Presidential Committee on Information Literacy: Final Report, released January 10, 1989: To be information literate, a person must be able to recognize when information is needed and have the ability to locate, evaluate, and use effectively the needed information. Producing such a citizenry will require that schools and colleges appreciate and integrate the concept of information literacy into their learning programs and that they play a leadership role in equipping individuals and institutions to take advantage of the opportunities inherent within the information society. Ultimately, information literate people are those who have learned how to learn. They know how to learn because they know how knowledge is organized, how to find information, and how to use information in such a way that others can learn from them. They are people prepared for lifelong learning, because they can always find the information needed for any task or decision at hand" [90].

**Institution:** Can also refer to facility or organization, where applicable.

**Integrated library system (ILS):** An ILS is library automation software that provides centralized management and processes for different types of libraries and library activities such as acquisition, cataloguing, circulation, administration, reporting and user records

**Interlibrary loan:** Interlibrary loan is a mechanism for borrowing or lending original materials between cooperating libraries.

**Librarian:** A health information professional who has obtained an MLIS or MIS. The MLIS (Master of Library and Information Studies) or MIS (Master of Information Studies) requires at a minimum an undergraduate degree as a point of entry into this field of study; it is a professional degree attained, but not licensed, through successful completion of a master's degree.

**Library:** A comprehensive selection of services and resources that are tailored to meet the information needs of a specific user group, organized for ease of access and under the direction of a health information professional.

**Library technician:** A health information professional who has obtained a Library Technician Diploma. The Library Technician Diploma requires a minimum of a high school diploma as a point of entry into this programme.

**Outreach:** Outreach is taking library services beyond the institution and its traditional users to the broader community.

**Patient education:** “The teaching or training of patients concerning their own health needs” [91].

**Proxy server:** A proxy server is a server that sits between a client application, such as a Web browser, and a real server. It intercepts all requests to the real server to see if it can fulfill the requests itself. If not, it forwards the request to the real server.

**User:** Any individual or library receiving service from Library and Information Services. Users may be internal or external to the facility.

**Virtual private network (VPN):** A VPN extends a private network across a public network, by maintaining sophisticated security that enables users to send and receive data across shared or public networks as if their computing devices were directly connected to the private network.

**Virtual space:** A compelling online presence or web site able to host content and provide access to eResources enhanced with social media to boost exposure and reputation as well as market services to potential users
